# Predictors of Diabetic Retinopathy in Type 2 Diabetes: A Cross-Sectional Study

**DOI:** 10.3390/biomedicines12081889

**Published:** 2024-08-19

**Authors:** Adriana Ivanescu, Simona Popescu, Radu Ivanescu, Monica Potra, Romulus Timar

**Affiliations:** 1 Second Department of Internal Medicine, “Victor Babes” University of Medicine and Pharmacy, 300041 Timisoara, Romania; adriana.ivanescu@umft.ro (A.I.); monica.rusu@umft.ro (M.P.); timar.romulus@umft.ro (R.T.); 2 Opticlass Ophtalmology Clinic, 300012 Timisoara, Romania; 3 Department of Diabetes, “Pius Brinzeu” Emergency Hospital, 300723 Timisoara, Romania

**Keywords:** type 2 diabetes mellitus, diabetic retinopathy, risk factors, quality of life

## Abstract

Background: Type 2 diabetes mellitus (T2DM) represents one of the most impacting health issues of the modern era, as it is associated with an extensive range of comorbidities. Diabetic retinopathy (DR) is one the utmost severe diabetes complications as it is one of the major causes of vision loss among these patients. Our present research aims to evaluate the most frequent risk factors related to the occurrence of DR in T2DM patients. Method: This study consisted of a research group of 302 participants, priorly diagnosed with T2DM, that were evaluated for the most important risk factors related to the occurrence of DR. Results: Patients had a median age of 64 years, 48% of them being women, with a 12-year median duration of DM and presenting a deficient glycaemic control echoed by a median HbA1C value of 7.5%. From the total number of participants, the total prevalence of DR in different stages of severity was 34.8% with a 95% CI. Statistically significant values were found regarding DM duration (*p* = 0.007), HbA1c > 7.2% (*p* = 0.001) and patients aged over 67 years (*p* = 0.0035), all these parameters being directly linked to DR. Conclusions: Older patients with T2DM that have a longer disease duration and simultaneous comorbidities present a higher risk of DR development, consequently a stringent management of these pathologies is needed.

## 1. Introduction

Type 2 diabetes mellitus (T2DM) is a chronic and progressive cardio-metabolic disorder, ranking among the primary causes of morbidity, disability, and mortality worldwide. According to the International Diabetes Federation (IDF), the global prevalence of diabetes mellitus (DM) was 463 million in 2019 and is projected to reach 700 million by 2045 [1]. Diabetic retinopathy (DR), a prevalent and severe microvascular complication of DM, is a leading cause of blindness among the adult population. In recent years, we noticed an important increase in both the prevalence of DR and the rates of blindness caused by it, representing a significant threat to the quality of life for diabetic patients. With a growing global population, longer lifespans for those with DM, and lifestyle changes elevating DM risk, the medical and economic burden of DR is expected to continue rising. This situation requires the urgent need for comprehensive eye care and extensive treatment strategies to address this pressing public health challenge [2,3].

Despite the decrease in the proportion of diabetic individuals developing proliferative diabetic retinopathy (PDR) and severe vision loss from 1980 to 2008 in populations with better diabetes control, there has been a significant increase in the prevalence of visual impairment and blindness caused by DR between 1990 and 2015. This trend, highlighted by the Vision Loss Expert Group of the Global Burden of Disease Study, is primarily attributed to the rising prevalence of T2DM [4].

Routine eye exams and timely interventions, coupled with systemic management of blood glucose, hypertension, and dyslipidaemia, are vital for reducing vision loss related to DM. Advances in technology and the rise of biologic treatments offer promising new and effective options to achieve this purpose. Nevertheless, the most crucial strategy for preventing ocular complications in diabetic patients remains maintaining strict glycaemic control [5,6].

Additional risk factors for DR encompass dyslipidaemia, high body mass index (BMI), puberty, pregnancy, and cataract surgery. Yet, clinical investigations in diabetic patients have unveiled substantial diversity in the onset and severity of DR, not fully attributable to all these established risk factors. Clinicians acknowledge that not all individuals with poor glycaemic control or uncontrolled blood pressure develop DR. Conversely, some patients with well-maintained glycaemic levels and no hypertension might still experience its onset. Considering these aspects, genetic factors might be involved in this variability. Still, further research is needed to confirm their impact on this pathology, with a future better understanding of DR [4].

The purpose of this this study was to evaluate the main conventional risk factors associated with the occurrence of DR in patients with T2DM.

## 2. Materials and Methods

### 2.1. Study Design and Population

This cross-sectional, non-interventional study was conducted at the Outpatient Diabetes Care Facility Centre of the Pius Brinzeu County Emergency Hospital Timisoara between 21 May 2024 to 24 June 2024. From a total of 573 patients that attended their prescheduled visits at the Diabetes Care Center, we enrolled a final number of 302 participants (145 females, 157 males), while 157 patients declined to participate in this research for personal reasons and 114 of them did not meet the inclusion criteria or were excluded due to the presence of any of the undermentioned general or ocular health status. Exclusion criteria referred to severe cognitive impairment or psychiatric disorders that prevented patients from providing informed consent, other medical pathologies that required hospitalisation on the duration of the study or institutionalised patients. Also, exclusion criteria referred to ophthalmologic diseases that impede proper fundus examination (advanced cataract and significant vitreous haemorrhage) or other retinal diseases that interfere with a proper classification of diabetic retinopathy by altering retinal structure (high myopia, retinal detachment, significant epiretinal membranes). Informed consent was obtained from all participants, none of them being involved in the development of this study. This research process was performed according to the Declaration of Helsinki (2013 version) and was approved by the Ethics Committee of the Emergency County Hospital “Pius Brinzeu” Timișoara (approval no. 467, approved on 20 May 2024)

Selected patients were above 18 years of age and have been priorly diagnosed with T2DM. Study participants had a median age of 64 years [57; 70] and a median T2DM duration of 12 years [6; 17]. Patients underwent the standardised general evaluation that is performed by their physician at each visit and also a comprehensive eye exam performed by an ophthalmologist. Study protocol included the assessment and evaluation of demographic characteristics, anthropometric indexes, diabetes mellitus profile and metabolic control, general comorbidities, ocular diabetes complications and smoker status.

### 2.2. Data Collection and Medical Assessment

Demographic data regarding all patients were obtained from their medical chart. Biological parameters were assessed by performing a full blood panel for all patients during their scheduled visit in the Diabetes Care Center. Diagnosis of T2DM was priorly made by the presence of a fasting plasma glucose level > 7.0 mmol/L (126 mg/dL), a 2-h post load plasma glucose > 11.1 mmol/L (200 mg/dL) or HbA1c level above 6.5% (48 mmol/mol). Disease duration was defined as the number of years passed from DM diagnosis to the date of inclusion in this study.

Anthropometric data, such as height and weight, were measured for each patient. BMI was calculated using the metric system, by dividing the weight measured in kg by squared height measured in meters. According to World Health Organization (WHO), we considered patients with BMI above 25 kg/m^2^ to be overweight, while those with BMI equal or above 30 kg/m^2^ were diagnosed with obesity.

Blood pressure was measured for all participants using an aneroid sphygmomanometer. Hypertension was considered at a systolic blood pressure above or equal to 140 mmHg and/or diastolic blood pressure values above or equal to 90 mmHg. Presence of cardiovascular disease (CVD), meaning coronary heart disease, strokes or peripheral arterial disease was noted according to patients’ medical history.

Diabetic neuropathy was diagnosed by performing nerve conduction velocity (NCV) and with the usage of the Michigan Neuropathy Screening Instrument (MNSI). A positive diagnosis was considered with a MNSI clinical score above 2.5, a questionnaire score above 7 or overall score above 9.5 and a NCV less than 40 m/s.

Chronic kidney disease (CKD) was diagnosed by analysing the creatinine-based estimate of glomerular filtration rate (eGFR) and albumin/creatinine ratio (ACR).

Presence of dyslipidaemia was noted according to lipid panel values. Smoker status was self-reported.

A comprehensive fundus examination with pupil dilation was performed on all patients by a trained ophthalmologist. Presence of cataract or previous cataract surgery with intraocular lens implantation (IOL) was noted. Examination of the central and peripheral retina included assessing the following findings: microaneurysms or retinal haemorrhage, cotton wool spots, hard exudates and macular oedema. Regarding retinal vasculature, the following aspects were evaluated: venous beading (VB), intraretinal microvascular abnormalities (IRMA) and neovascularisation of the optic disc (NVE) or elsewhere in the retina (NVE). Those findings were the basis for DR and diabetic maculopathy (DME) diagnosis, according to the Early Treatment Diabetic Retinopathy Study Design (ETDRS) guidelines. DR was classified as follows: nonproliferative diabetic retinopathy (NPDR) with mild NPDR (at least one microaneurysm), moderate NPDR (microaneurysms or haemorrhages, exudates, cotton wool spots, VB, IRMA) and severe NPDR (microaneurysms or haemorrhages present in all four retinal quadrants, IRMA in at least one quadrant and VB in two or more quadrants) and proliferative diabetic retinopathy (PDR) which was defined by the presence NVE and preretinal or vitreous haemorrhage. DME was considered when exudates or retinal thickening were found within at least one disc diameter from the fovea. Intraocular pressure (IOP) was measured for all patients after the instillation of an anaesthetic and a drop of fluorescein dye by using Perkins handheld applanation tonometer.

### 2.3. Statistical Analysis

Statistical data were gathered and analysed by using MedCalc ^R^ Statistical Software, the 22.016 version, developed by MedCalc Software Ltd. in Ostend, Belgium. This software may be accessed at https://www.medcalc.org (accessed on 30 May 2024) and was used in May 2024. The analysed cohorts were depicted by using median and interquartile range (continuous variables with non-parametric distribution) and minimum and maximum values. The categorical variables are presented by absolute and relative (percentages) frequencies. In order to evaluate the statistical significance of differences in central tendency indicators between the evaluated groups, we used unpaired Student’s *t*-test for comparing the arithmetic means of the parametric variables between two groups, Mann–Whitney-U tests for comparing two medians, ANOVA test to analyse the variation of the mean values between more than two groups, respectively, Kruskal–Wallis test to study the variation of the median values between more than two groups.

Regarding the assessment of statistically significant differences between percentages, we performed chi-square tests (for comparison of two proportions) and chi-square for trend (for comparison between more than two proportions). Spearman’s correlation coefficient was used to evaluate the strength and direction of the associations between the numerical variables.

To investigate the adjusted strength, direction and significance of the associations between more than two numerical variables, multivariate logistic regression models were built, while to evaluate the association between numerical (independent) and dichotomous (dependent) variables, multivariate logistic regression models were applied.

“Receiver-Operating Characteristics” (ROC) analyses were conducted in order to appraise the performance of continuous variables as predictive factors for a dichotomous outcome. The prediction threshold was considered the Youden’s index (the point which maximizes the sum of sensitivity and specificity of the test), while the evaluated variables were considered as valid predictors if the area under the ROC curve was significantly higher than 0.5.

The sample size was a priori calculated to provide a confidence level (1-a) of 0.95 in parallel with a statistical power (1-b) of 0.80 for the study’s primary outcome.

In the present research, we provided a 95% confidence interval and considered the threshold of statistical significance at a *p*-value lower than 0.05.

## 3. Results

This study included a final number of 302 participants, with a median age of 64 years, 48% being women (145 individuals). Patients had a median duration of DM of 12 years, with an inadequate glycaemic control reflected by the median HbA1C value of 7.5%. Most of the study population presented several comorbidities like diabetic polyneuropathy, hypertension, CVD, CKD and dyslipidaemia. The main characteristics of the whole study group are exhibited in Table 1.

**Figure 1:**
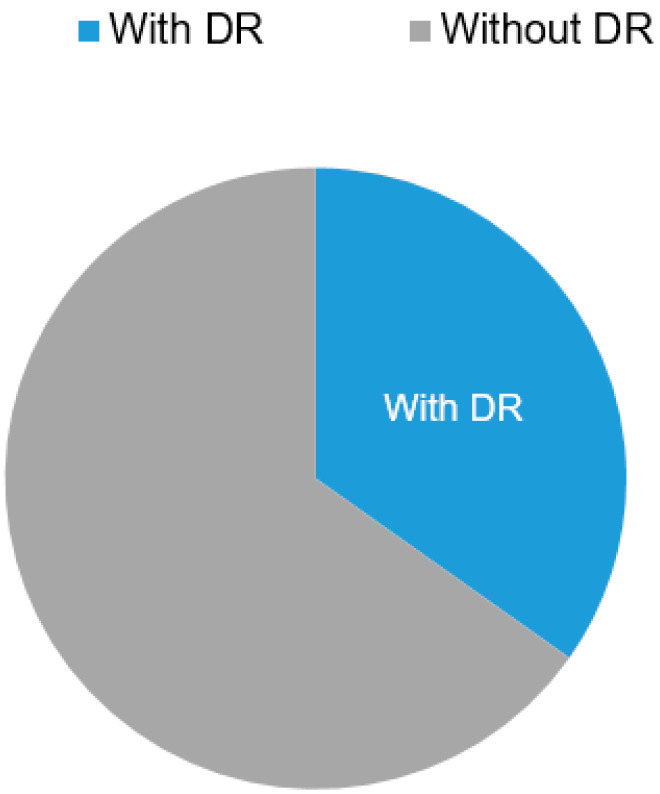
The prevalence of DR.

**Figure 2:**
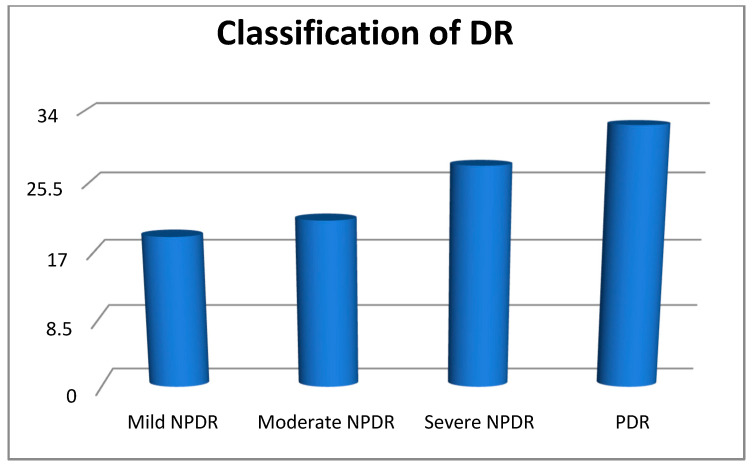
The classification of DR.

In our study group, DR was found in 105 patients, representing a prevalence of 34.8% [28.4–42.1%], 95% CI (Figure 1). According to the ophthalmologic evaluation, most of these patients presented proliferative diabetic retinopathy (PDR) 32.4%, 27.6% presented severe nonproliferative diabetic retinopathy (NPDR), 21% individuals had moderate NPDR, while only 19% of the study group presented a mild stage of NPDR (Figure 2).

In diabetic patients with DR (Table 2) gender distribution was almost equal, with 47.6% women, patients presented a mean age of 66 years old. The mean DM duration was 15 years, while HbA1C median value was 8% [7.5–8.8%]. The majority of these patients were overweight (mean BMI 28.3 Kg/m^2^) and presented mostly cardiovascular comorbidities (hypertension 78.1% and CVD 86.7%), but also diabetic polyneuropathy (71.4%) and dyslipidaemia (59%). Presence of DME was found in 18.1% of patients with DR, while intraocular pressure was within normal range in this subgroup (mean IOP 15.5 mmHg). Smoking status was self-reported among 25.7% of patients.

Regarding the severity of DR, a higher HbA1c value was associated with a more severe DR (from 7.1% in mild NDPR to 8.9% in PDR; *p* < 0.0001). In addition, a longer DM duration was associated with a more severe DR (from 8 years in mild NPDR to 20 years in PDR; *p* < 0.0001). No significant association between patients age and the severity of DR was found. The relationship between the severity of DR and studied parameters is presented in Table 3.

**Figure 3:**
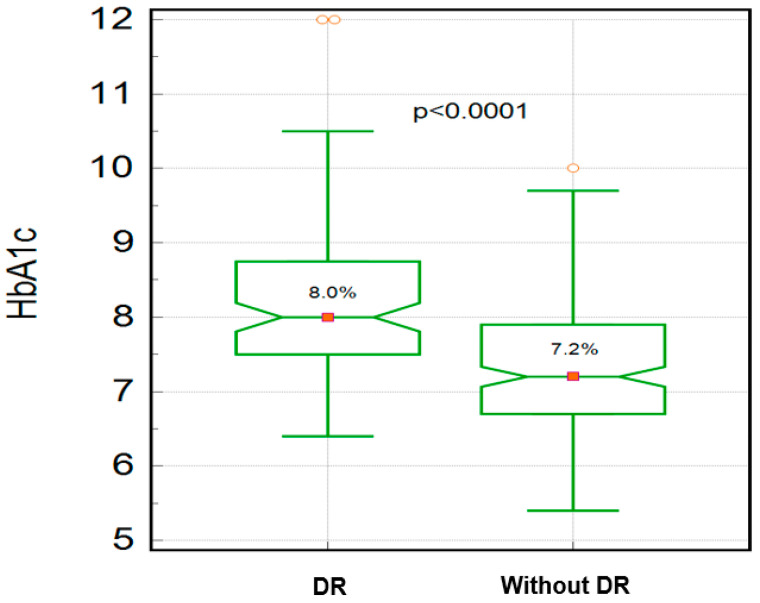
The median value of HbA1c in patients with DR vs. without DR.

**Figure 4:**
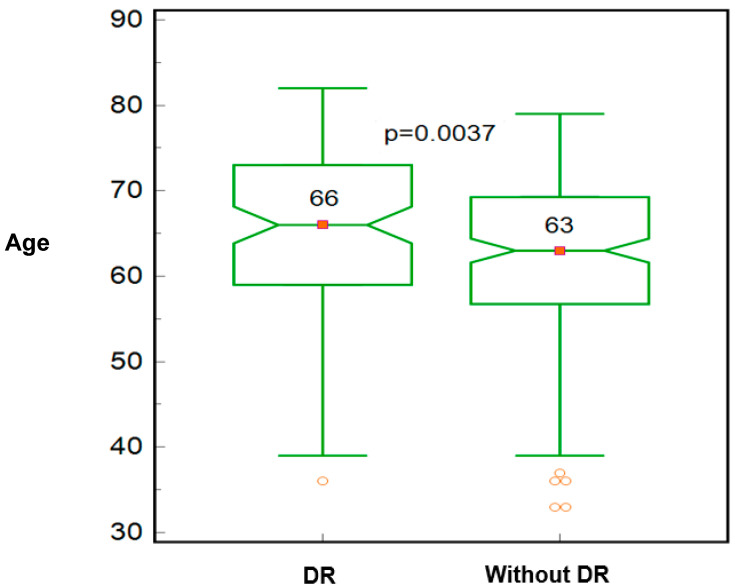
Median age value for patients with DR vs. without DR.

The presence of DR was associated with a significantly higher diabetes duration (15 vs. 12 years; *p* = 0.0076; Mann–Whitney test), HbA1c (8.0 vs. 7.2 percentage points; *p* < 0.0001; Figure 3), BMI (28.3 vs. 27 kg/m; *p* = 0.0064) and age (66 vs. 63 years; *p* = 0.037; Figure 4). No significant association was found between DR and IOP values, as illustrated in Table 4.

Considering the fact that the majority of the patients included in this study are elderly, we also evaluated the presence of cataract, as it represents the most frequent ocular pathology found among this age group. No significant differences were found between patients with vs. without DR (Table 5).

To evaluate the adjusted impact of HbA1c, age and DM duration on the development of DR, we built a multivariate logistic regression model. In this model, we identified that increases in HbA1c value and age significantly enhanced the risk of DR occurrence (Table 6). This model explained 25.29% of the factors involved in the development of DR in patients with T2DM.

**Figure 5:**
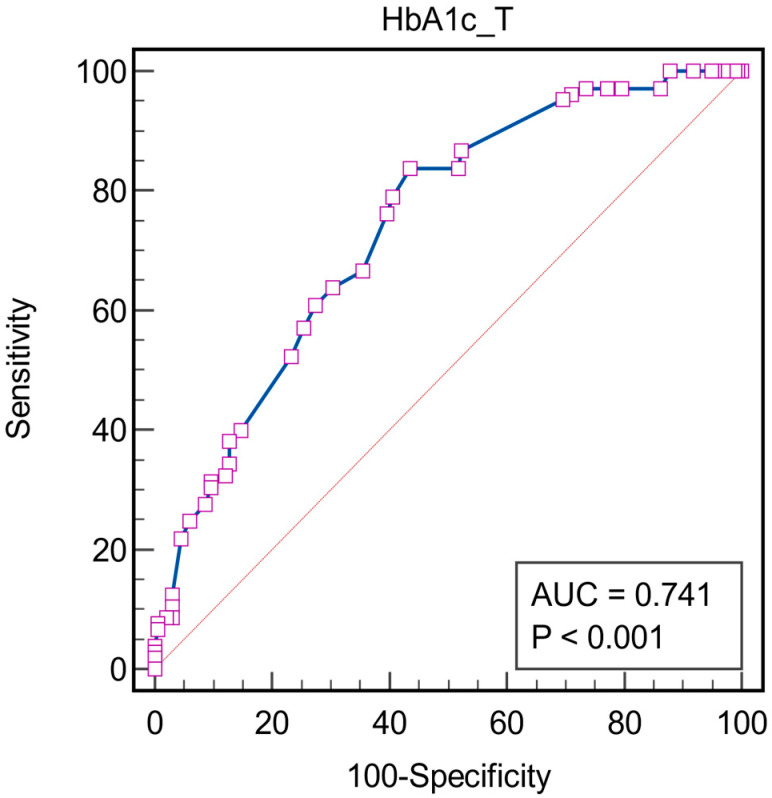
Receiver operating characteristics analysis for HbA1c as a predictor for DR. Associated criterion: HbA1c > 7.2%, AUC = 0.741; Youden index J: 0.4015 95%, CI: 0.688 to 0.790; Sensitivity: 83.81%, z statistic = 8.417; Specificity: 56.36%, *p* < 0.0001. The squares represent individual data points, the blue line represents the ROC curve and the red diagonal line is the reference line that represents a model with no discriminative ability.

To evaluate the prediction performance of HbA1c in the development of DR, ROC analysis was performed. The results pointed out that HbA1c value is a valid predictor for the development of DR (AUROC = 0.741; *p* = 0.001), having the best prediction threshold, according to the Youden’s method at a HbA1c value higher than 7.2%, corresponding to a sensitivity of 83.81% and associated specificity of 56.35% (Figure 5).

**Figure 6:**
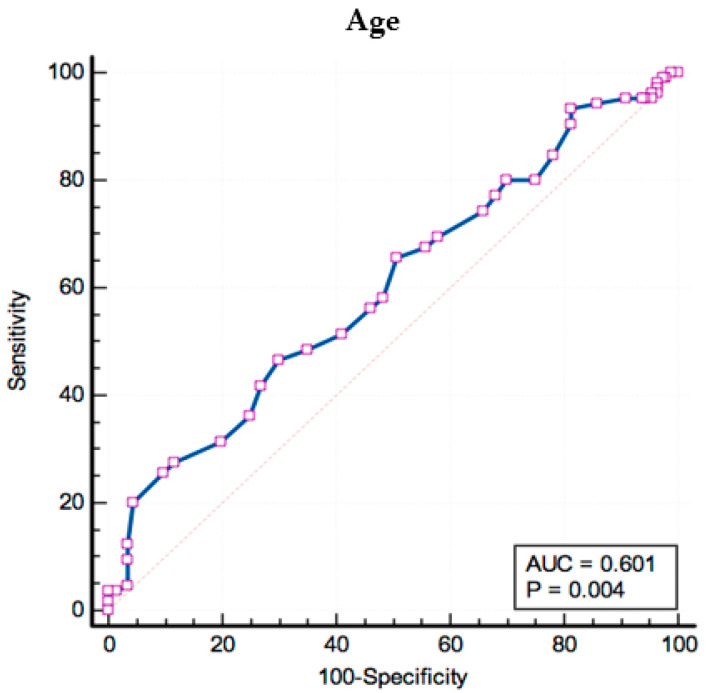
Receiver operating characteristics analysis for patient’s age as a predictor for DR. Associated criterion: Age > 67 years, AUC = 0.601; Youden index J: 0.1672 95%, CI: 0.544 to 0.657; Sensitivity: 46.67%, z statistic = 2.916; Specificity: 70.05%, *p* = 0.0035. The squares represent individual data points, the blue line represents the ROC curve and the dotted red diagonal line is the reference line that represents a model with no discriminative ability.

Another valid predictor for the development of DR in patients with Type 2 DM was patient’s age, with a threshold of > 67 years, corresponding to a sensitivity of 46.47% and specificity of 70.05% (AUROC = 0.601; *p* = 0.004; Figure 6).

**Figure 7:**
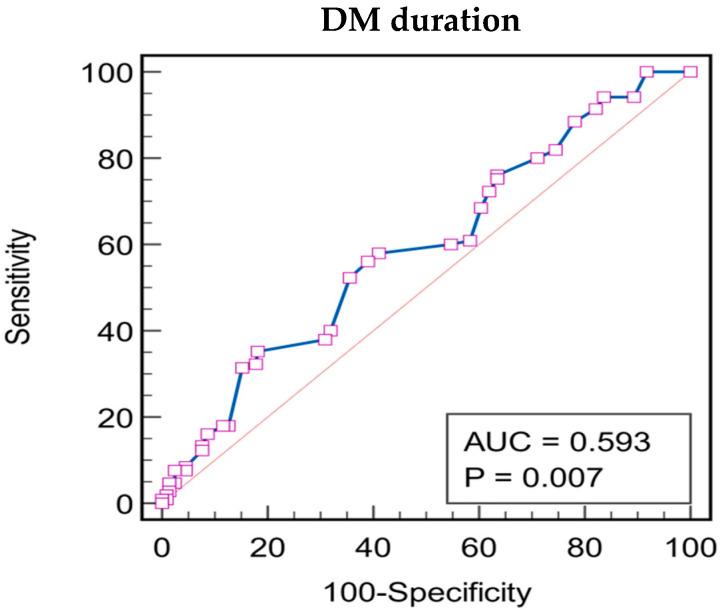
Receiver operating characteristics analysis for patient’s DM duration as a predictor for DR. Associated criterion: DM duration > 13years, AUC = 0.593; Youden index J: 0.1710 95%, CI: 0.535 to 0.649; Sensitivity: 56.19%, z statistic = 2.719; Specificity: 60.91%, *p* = 0.0065. The squares represent individual data points, the blue line represents the ROC curve and the red diagonal line is the reference line that represents a model with no discriminative ability.

Regarding DM duration, as stated by the ROC curve parameters (AUROC = 0.593, *p* = 0.007), DM duration over 13 years was associated with a sensitivity of 56.19% and specificity of 60.91% (Figure 7).

**Figure 8:**
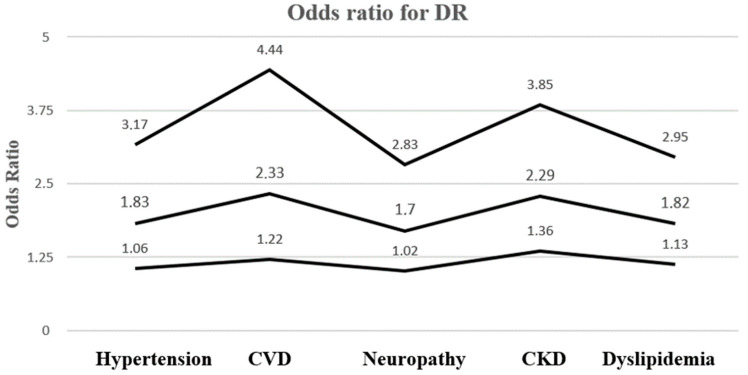
Odds ratio for DR development. CVD, cardiovascular disease; CKD, chronic kidney disease.

Most T2DM patients, including the population of this study, exhibit a various number of comorbidities that significantly impact their quality of life by also subsequently increasing their potential risk of developing DR. As shown in Table 7, the presence of other diabetes complications or comorbidities (hypertension, CVD, CKD and dyslipidaemia) were associated with a higher risk of DR occurrence (Figure 8), while no significant differences were found regarding polyneuropathy or smoking.

## 4. Discussion

This present study aimed to evaluate the main risk factors involved in the development and progression of DR among patients with T2DM. From all patients included in this study, 34.8% presented DR in different stages of severity. These results align with recent literature, which reports a median prevalence of this disease at 27.9% (range: 22–37%) among patients with type 2 diabetes [7].

An important fact to be noted regarding DR prevalence is outlined by a study conducted by Markle et al. that highlighted a current increase in DR prevalence among younger diabetic patients, while older ones exhibit a slight decrease in its occurrence [8]. Taking this recent research into consideration, and the fact that our study group was comprised mainly of elderly patients (median age 64 years old), results regarding prevalence could increase if the study group was extended to a younger category of T2DM patients.

Facing a global ageing population [9], the concept of multimorbidity has gained increasing interest among primary care physicians [10], especially regarding T2DM patients, as they are at a higher risk of presenting multiple co-occurring conditions [11]. In our study group, most of the patients presented more than one comorbidity alongside DM. Our focus was on observing the main systemic pathologies such as hypertension, CVD, CKD and dyslipidaemia, diseases that are known to be highly prevalent in the elderly population with a significant impact on diabetes-related ocular complications such as the vision-threatening DR. Regarding polyneuropathy, one of the most frequent chronic DM complications, we obtained a strong correlation between DR occurrence and the presence of polyneuropathy among T2DM patients as stated in current literature [2,12]. We found that the above mentioned pathologies (hypertension, CVD, polyneuropathy and dyslipidaemia) are highly prevalent among our general study group, but more importantly a high prevalence was observed regarding the subpopulation represented by patients with DR. Hypertension and CVD had one of the highest predictive values in our study regarding probability of DR occurrence, similarly to current literature data [13,14]. Identifying a positive correlation between polyneuropathy, dyslipidaemia and DR has been the purpose of multiple international studies [15,16], our results being superposable with current literature.

Regarding other ocular pathologies, we observed the presence of cataract or previous cataract surgery with IOL implant, IOP values and last but not least DME presence. Cataract is known to be more frequent among diabetic patients [17], with 10-year incidence of cataract surgery being 24.9% among this category of patients according to the Wisconsin Epidemiologic Study of Diabetic Retinopathy [18]. In our research, 20.2% of T2DM patients without DR and 21% of patients with DR presented cataract or previous cataract surgery. IOP values obtained in our group were within normal range with a median value of 15 mmHg in both patients with or without DR, still, positive correlations are known between higher IOP and presence of glaucoma among diabetic patients [19]. DME can virtually occur in any stage of DR, but remains more frequent among patients with PDR [20]. Population-based studies show a reported prevalence of DME of up to 12.8% [21], while our research obtained similar results, more precisely 18.1% prevalence among our studied population.

Our research population consisted of mainly overweight patients. BMI had a median value of 27 kg/m^2^ [25; 30.8] across the entire study group, with a higher median value of 28.3 kg/m^2^ [25.7; 31] among patients who also had DR. This is lower than the prevalence of overweight patients reported in the current literature for those with T2DM, which ranges from 31.2% to 67.8%, a condition known to be linked to DR progression [22,23]. These differences could be attributed to the fact that our study group mainly consisted of elderly patients with longer diabetes duration and poor glycaemic control, factors that could suggest subsequent weight loss due to uncontrolled DM and the presence of metabolic complications [23]. General DM management typically focuses on weight loss for overweight and obese patients; however, some precautions should be taken when applying these measures in the elderly diabetic population, as recent data suggest fewer benefits from extensive weight control in this age group [24].

Behavioural aspects like smoking, alcohol use or practicing physical activity are key factors in maintaining a healthy lifestyle in general population but more so in diabetic patients. Improving these negative patterns, diabetes incidence is reduced among the general population, while it significantly decreases the risk of complications, especially CVD among diabetics [25]. Our study has focused on smoking assessment, as it is considered one of the most avertible causes of death globally. Results were noted after patients self-report, illustrating that smoking was more prevalent in DR subpopulation (25.7%) than in the whole study group (19.5%), data that aligned with current literature [26]. In our research, we only considered active smokers, as nicotine intake contributes to impairment in insulin activity and has a subsequent negative effect on glycaemic control. However, data are inconclusive regarding a direct impact of smoking on the progression of DR [26,27].

Our research strengths consists of a relatively large study population that we consider accurately reflects health status among elderly T2DM patients in our country, but we also weigh the need to include a younger demographic group in further research, as early-onset T2DM patients seem to develop systemic complication earlier and more aggressively [28].

All patients included in this study were subjected to a complete fundus examination with prior pupil dilation in order to evaluate the presence of DR and DME. Still, this method remains slightly subjective and consequently can lead to underdiagnosis of the extend of retinal disease and presence of macular thickening [29]. We consider a more extended ophthalmologic evaluation of these patients is needed, utilising fundus photography, but more important optical coherence tomography (OCT). OCT and the more novel OCT-angiography offer high-resolution images of retinal structures and vasculature in order to properly identify diabetes related ophthalmic complications, aiding ophthalmologists in establishing a proper treatment protocol and a precise follow-up [30,31,32].

The not so new concept of empowerment, which according to WHO refers to a practice in which people gain control over their own actions regarding their health, has become prominent lately also among diabetic patients. This concept is considered to improve the patient–physician relationship, and ultimately determining patients to become more adherent to adequate medical conduct [33]. Nevertheless, in order to achieve an empowered status among diabetic population, more thorough patient education is due. Besides the strict management of the risk factors that has been the object of many studies, including the present one, patients should be more informed about possible DR symptoms like sudden or gradual vision loss, presence of floaters in the visual field, loss of central vision or impaired night vision. Lack of treatment adherence among diabetic patients is a very common issue; therefore, a more intensive patient education regarding their disease might overcome this barrier, ultimately improving their long-term quality of life [34].

## 5. Conclusions

The results obtained from our present research confirm current literature data which state that the most important risk factors regarding T2DM evolution and management involved in the occurrence of DR are represented by poor glycaemic control and longer DM duration, while the most impacting comorbidities are hypertension and CVD, CKD and dyslipidaemia. Early detection and management of systemic risk factors can avert this DM complication. However, the primary and fundamental strategy for preventing ocular complications in diabetic patients continues to be the maintenance of a tight glycaemic control.

## Figures and Tables

**Table 1:** Characteristics of the studied group.

Variable	Value
Number of patients	302
Women Nr (%) ^a^	145 (48.0%)
Age (years) ^b^	64 [57; 70]
DM duration (years) ^b^	12 [6; 17]
BMI (Kg/m^2^) ^b^	27 [25; 30.8]
HbA1c (%)^b^	7.5 [7; 8]
Mean IOP (mmHg) ^b^	15 [13; 17]
IOP right eye (mmHg) ^b^	14 [12; 16]
IOP left eye (mmHg) ^b^	16 [14; 18]
Cataract or IOL implant Nr (%) ^a^	61 (20.2%)
DR Nr (%) ^a^	105 (34.8%)
Hypertension Nr (%) ^a^	212 (69.5%)
CVD Nr (%) ^a^	236 (78.2%)
Diabetic polyneuropathy Nr (%) ^a^	191 (63.3%)
CKD Nr (%) ^a^	84 (27.8%)
Dyslipidaemia Nr (%) ^a^	150 (49.7%)
Smoker status Nr (%) ^a^	59 (19.5%)

Notes: ^a^ categorical variables are presented by absolute frequency (percentage) in the sample. ^b^ continuous variables (with non-Gaussian distribution) are indicated by their median (interquartile range); Abbreviations: DM, diabetes mellitus; BMI, body mass index; HbA1C, haemoglobin A1c; IOP, intraocular pressure; IOL, intraocular lens; DR, diabetic retinopathy; CVD, cardiovascular disease; CKD, chronic kidney disease.

**Table 2:** Characteristics of patients with DR.

Variable	Value
Number of patients	105
Women Nr (%) ^a^	50 (47.6%)
Age (years) ^b^	66 [59; 73]
DM duration (years) ^b^	15 [7.9; 20]
BMI (Kg/m^2^) ^b^	28.3 [25.7; 31]
HbA1c (%) ^b^	8 [7.5; 8.8]
Mean IOP (mmHg) ^b^	15.5 [14; 17.5]
IOP right eye (mmHg) ^b^	15 [14; 17]
IOP left eye (mmHg) ^b^	16 [14; 18]
Cataract or IOL implant Nr (%) ^a^	22 (21%)
DME Nr (%) ^a^	19 (18.1%)
Hypertension Nr (%) ^a^	82 (78.1%)
CVD Nr (%) ^a^	91 (86.7%)
Diabetic polyneuropathy Nr (%) ^a^	75 (71.4%)
CKD Nr (%) ^a^	41 (39.1%)
Dyslipidaemia ^a^	62 (59.0%)
Smoker status Nr (%) ^a^	27 (25.7%)
Argon laser photocoagulation Nr (%) ^a^	56 (53.3%)

Notes: **^a^** categorical variables are presented by absolute frequency (percentage) in the sample. ^b^ continuous variables (with non-Gaussian distribution) are indicated by their median (interquartile range). Abbreviations: DM, diabetes mellitus; BMI, body mass index; HbA1C, haemoglobin A1c; IOP, intraocular pressure; IOL, intraocular lens; DME, diabetic macular oedema; DR, diabetic retinopathy; CVD, cardiovascular disease; CKD, chronic kidney disease.

**Table 3:** Characteristics of people with DR depending on the severity of DR.

Parameter	Mild NPDR	Moderate NPDR	Severe NPDR	PDR	*p* *
HbA1c (%) ^b^	7.1 [7; 7.45]	8 [7.5; 8.6]	8 [7.45; 8.2]	8.9 [8; 9.1]	<0.0001
DM duration (years) ^b^	8 [5; 10]	5 [3; 11.5]	15.5 [12.5; 20]	20 [15; 23]	<0.0001
Age (years) ^b^	68 [60.5; 72]	69 [58.2; 75.7]	64.5 [56; 70.5]	64.5 [60; 73]	0.51

Notes: Numerical variables with non-Gaussian distribution are indicated by their median (interquartile range); Results are presented as median (interquartile range). *p*-value was calculated using Kruskal–Wallis test. Abbreviations: HbA1C, haemoglobin A1c; DM, diabetes mellitus; NPDR, nonproliferative diabetic retinopathy. ^b^ Continuous variable; * ANOVA: Kruskal-Wallis test.

**Table 4:** Comparison between patients with and without DR.

Variable	With DR			Without DR			
	*n*	Median	Average Rank	*n*	Median	Average Rank	*p* ^a^
DM duration	105	15.0000	169.8524	197	12.0000	141.7183	0.0076
HbA1c	105	8.0000	199.0190	197	7.2000	126.1726	<0.0001
BMI	105	28.2828	170.2476	197	27.0000		0.0064
IOP OD	105	14.0000	121.7476	197	13.5000	109.2863	0.1530
IOP OS	105	16.0000	156.3095	197	16.0000	148.9365	0.4821
Age	105	66.0000	171.4619	197	63.0000	140.8604	0.0037

Notes: ^a^ Mann–Whitney test. Abbreviations: DM, diabetes mellitus; IOP, intraocular pressure.

**Table 5:** Comparison between patients with and without DR regarding associated cataract.

	DR	Without DR	*p* *	Chi-Squared
Cataract Nr (%) ^a^	22 (21%)	39 (19.8%)	0.8120	0.0565

Notes: * Chi-squared test. ^a^ Numerical values with non-Gaussian distribution. Results are presented in percentage from total. Abbreviations: DR, diabetic retinopathy.

**Table 6:** Predictors for DR development in patients with T2DM.

Variable	Odds Ratio	95% CI
HbA1c	2.6624	1.9309 to 3.6710
Age	1.0500	1.0184 to 1.0826
DM duration	1.0182	0.9859 to 1.0515

Nagelkerke R^2^ = 0.2529. Significance level *p* < 0.0001.

**Table 7:** The probability of DR occurrence depending on the presence of various diseases.

Comorbidity	OR	95% CI	Z Statistic	Significance Level
HTA	1.8375	1.0618 to 3.1797	2.174	*p* = 0.0297
CVD	2.3310	1.2221 to 4.4462	2.569	*p* = 0.0102
Polyneuropathy	1.7026	1.0215 to 2.8379	2.042	*p* = 0.0412
CKD	2.2943	1.3672 to 3.8502	3.144	*p* = 0.0017
Dyslipidaemia	1.8275	1.1299 to 2.9558	2.458	*p* = 0.0140
Smoker status	1.7740	0.9945 to 3.1647	1.941	*p* = 0.0522

## Data Availability

All available data can be provided upon request to the corresponding author.
